# NR4A1 Promotes LPS-Induced Acute Lung Injury through Inhibition of Opa1-Mediated Mitochondrial Fusion and Activation of PGAM5-Related Necroptosis

**DOI:** 10.1155/2022/6638244

**Published:** 2022-02-18

**Authors:** Pingjun Zhu, Junyan Wang, Wenjuan Du, Jun Ren, Ying Zhang, Fei Xie, Guogang Xu

**Affiliations:** ^1^Department of Respiratory Medicine, The Second Medical Center & National Clinical Research Center for Geriatric Diseases, Chinese PLA General Hospital, Beijing 100853, China; ^2^Chinese PLA General Hospital, Medical School of Chinese PLA, Beijing 100853, China; ^3^School of Pharmaceutical Sciences, Guangzhou University of Chinese Medicine, Guangzhou 510006, China; ^4^Laboratory of Radiation Injury Treatment, Medical Innovation Research Division, PLA General Hospital, Beijing 100853, China; ^5^Department of Cardiology, Shanghai Institute of Cardiovascular Diseases, Zhongshan Hospital, Fudan University, Shanghai 200032, China; ^6^Center of Pulmonary and Critical Care Medicine, Chinese PLA General Hospital, Beijing 100853, China; ^7^The Second Medical Center & National Clinical Research Center for Geriatric Diseases, Chinese PLA General Hospital, Beijing 100853, China

## Abstract

Mitochondrial dysfunction and necroptosis have been perceived as the primary molecular mechanisms underscoring acute lung injury. Meanwhile, nuclear receptor subfamily 4 group A member 1 (NR4A1) is considered a regulator of inflammation-related endothelial injury in lung tissue although the downstream molecular events remain elusive. In this study, we employed NR4A1^−/−^ mice to decipher the role of NR4A1 in the onset and progression of acute lung injury with a focus on mitochondrial damage and necroptosis. Our results demonstrated that NR4A1 was significantly upregulated in lipopolysaccharide- (LPS-) treated lung tissues. Knockout of NR4A1 overtly improved lung tissue morphology, inhibited inflammation, and reduced oxidative stress in LPS-treated lung tissue. A cell signaling study suggested that NR4A1 deletion repressed levels of PGAM5 and attenuated LPS-mediated necroptosis in primary murine alveolar epithelial type II (ATII) cells, the effects of which were mitigated by PGAM5 overexpression. Moreover, LPS-mediated mitochondrial injury including mitochondrial membrane potential collapse and mitochondrial oxidative stress was drastically improved by NR4A1 deletion. Furthermore, NR4A1 deletion preserved mitochondrial homeostasis through activation of Opa1-related mitochondrial fusion. Silencing of Opa1 triggered mitochondrial dysfunction in NR4A1-deleted ATII cells. Taken together, our data identified NR4A1 as a novel regulator of LPS-related acute lung injury through regulation of mitochondrial fusion and necroptosis, indicating therapeutic promises of targeting NR4A1 in the treatment of acute lung injury in clinical practice.

## 1. Introduction

Acute respiratory distress syndrome (ARDS), a diffused injury of the lung parenchyma evoked by pathological stresses including severe infection, anoxia, ischemia, trauma, and surgery [1, 2], is deemed one of the most common refractory complications with high mortality and mortality in critically ill patients [3, 4]. To date, effective therapy for ARDS is still lacking. Ample experimental findings have unveiled the pathogenesis of ARDS with beneficial effects from pharmacological and mechanical conditioning strategies [5]. In particular, a number of mechanisms have been suggested to contribute to ARDS including oxidative stress, inflammation response, and immunomodulatory disorders [6–8]. A better understanding of the interplay among these pathological factors should help to unveil novel therapeutic strategies for acute lung injury and clinical outcomes in patients with ARDS.

Given the important role of mitochondria in energy metabolism through consumption of nutrient and oxygen by way of tricarboxylic acid cycle and oxidative phosphorylation (OXPHOS), mitochondria serve as the powerhouse in cells [9]. Besides, a range of cellular physiological processes such as oxidative stress, calcium regulation, signaling transduction, cell movement, growth, proliferation, and death are controlled by mitochondria, especially in respiratory diseases including but not limited to ARDS. Mitochondrial-derived reactive oxygen species (ROS) has aroused considerable attention due to its dual regulatory role in ARDS [10]. In lipopolysaccharide- (LPS-) induced acute lung injury, mitochondrial APT production is reduced whereas levels of ROS are elevated, because of neutrophil accumulation in lung tissues [11]. Furthermore, mitochondrial ROS-triggered oxidative damage as well as mitochondrial apoptosis is noted in kidney tissues following LPS challenge [12]. Another independent study in a model of endotoxin-induced ARDS revealed a connection between mitochondrial bioenergetic dysfunction and impaired pulmonary ventilation [13]. These findings indicate a role of mitochondrial dysfunction in acute lung injury although precise molecular mechanisms are not fully understood. Recently, evidence has denoted that mitochondrial morphological alteration is preceded by mitochondrial injury [14, 15]. Increased mitochondrial fragmentations, as a result of enhanced mitochondrial fission and decreased mitochondrial fusion, are an early sign of mitochondrial membrane potential and mitochondrial ROS overload. Despite the well-recognized role of mitochondrial fission in LPS-triggered ARDS, it remains poorly understood whether and how mitochondrial fusion and changes in mitochondrial homeostasis partake in the development of ARDS.

Acute lung injury is featured by the death of functional cells such as alveolar epithelium through apoptosis or necrosis. Unlike necrosis, apoptosis is a regulated cell death program which can be intervened by pharmacologic inhibitors or genetical ablation. ARDS-related alveolar epithelium apoptosis has been well documented in experimental animal models or human samples [3, 16, 17]. Recently, a novel form of regulated cell death modality, namely, necroptosis, was found to be involved in the pathogenesis of ARDS through an undefined mechanism. Considering the relationship between mitochondrial damage and necroptosis, we wish to examine if necroptosis activation is secondary to mitochondrial morphological alteration and thus contributes to ARDS-related lung injury. NR4A1, a member of the NR4A nuclear receptor family of intracellular transcription factors, is a regulator of inflammation-related endothelial cells in lung tissues [18]. Furthermore, loss of NR4A1 promotes the phagocytic capacity of alveolar macrophages (AMs) and disrupts the host defense against invading bacteria, improving the outcome of *E. coli* pneumonia in mice, suggesting its potential role in ARDS etiology [19]. Various studies have noted a role for NR4A1 in the development of mitochondrial dysfunctions, such as mitochondrial oxidative stress, mitochondrial death, and mitochondrial fission in pathological positions including cardiac ischemia-reperfusion injury and fatty liver disease [20, 21]. To this end, we hypothesized that NR4A1 affects mitochondrial fusion to provoke necroptosis in the pathogenesis of ARDS.

## 2. Methods

### 2.1. Mouse Models of LPS-Induced Acute Lung Injury

All animal procedures employed here complied with the Guide for the Care and Use of Laboratory Animals. The study was approved by the Ethics Committee of the Chinese PLA General Hospital, Beijing, China. NR4A1^−/−^ mice were generated according to previously described [22]. They were housed in a cooled room (21 ± 2°C) with 12/12 h light cycles, relative humidity of 40–60%, and food and water ad libitum. All experimental mice were sex- and age-matched (*n* = 6/group). An ALI animal model was induced using an intratracheal injection of LPS (5 mg/kg) for 24 hours as previous described [23]. The Sham group was injected with PBS for 24 hours. All mice were sacrificed to collect BAL fluid and lung tissues.

### 2.2. Histological Analysis

Lung tissues were fixed in 4% fixative (Sigma-Aldrich) and embedded in paraffin blocks subsequently. 5 *μ*m thick slices were made, and hematoxylin and eosin (H&E, Sigma-Aldrich) were used to get specimen stained. The area of interest was measured using the ImageJ software.

### 2.3. Lung Wet to Dry (W/D) Ratio

The W/D ratio was used to estimate the severity of edema. The lung tissues were weighed after excising the left lung and removing blood. An 80°C oven was used to get the specimen dried for 4 days until an invariant weight.

### 2.4. Cell Culture and Treatment

As previously described, the excised lung tissues were incubated in 2.4 U/ml Dispase II (Sigma) at 37°C for 1 hour to get dispersed ATII cells [24]. Then, get the admixture filtered via a sterile stainless-steel mesh to get separated cells from residual tissues. Next, centrifuge cells and decant enzyme solution. Primary ATII cells were incubated in DMEM (HyClone, USA) containing 10% fetal bovine serum (FBS, HyClone, USA) at 37°C in a humidified incubator with 5% CO_2_. Primary ATII cells were cocultured with LPS (1 *μ*g/ml, Sigma-Aldrich, USA) for 24 hours to induce acute lung injury [25].

### 2.5. Construction of PGAM5 Overexpression Vector

PGAM5 cDNA was cloned to pShuttle-CMV. Recombinant adenovirus plasmid pShuttle-AdPGAM5 was produced in the BJ5183 strain. Viral packaging and purification, viral titer determination, and infection inspection were referred to as the method of Nature Protocols. Adenovirus transfection was conducted as our previously described [26]. The overexpression efficiency was verified by Western blot.

### 2.6. Opa1 siRNA and Transfection

The Opa1 siRNA kit was obtained from Sangon Biotech (Shanghai, China), and a specific method was operated based on the manufacturer's commands. The disorderly interfering siRNA was served as an experimental negative comparison. Opa1 siRNA and negative control siRNA were diluted in RNase-free water to obtain a 20 *μ*M raw solution and stored at −20°C. Opa1 siRNA and negative control siRNA were diluted in different proportions with Opti-MEM medium without serum. X-tremeGENE siRNA transfection reagent (Roche, Switzerland) was diluted with Opti-MEM medium and placed at 26°C for 5 minutes. Opa1 siRNA and negative control siRNA were mixed with an X-tremeGENE siRNA transfection reagent prior to incubation at 26°C for 20 minutes. The siRNA's final concentration was 150 nM. Through transfection for the first 24 hours, cells were challenged with LPS for the second 24 hours and then were collected for subsequent experimentation.

### 2.7. RNA Isolation and Quantitative Real-Time PCR

As previous study described, total RNA was isolated with a TRIzol reagent (Invitrogen, 15596026) from murine lung tissues or LPS-treated cells [27]. One microgram of total RNA was used for cDNA synthesis (Invitrogen). LightCycler 480 II system (Roche) was used for quantitative real-time PCR.

### 2.8. Immunoblotting

By using electrophoresis on a 4–20% SDS-polyacrylamide gel, equal amounts (20–35 *μ*g) of total protein were subdivided and subsequently transferred onto Immobilon-P PVDF membrane (EMD Millipore, IPV00010). Given the better separation protein efficiency of gradient gel, we intended to cut the membrane into small pieces to blot 2-3 proteins. In summary, membranes were pretreated with the Pierce^™^ reversible protein stain kit for PVDF membranes and were spitted into several pieces according to the predicted molecular sizes of target proteins, with a pertained Spectra Multicolor Broad Range Protein Ladder (Thermo Fisher Scientific, 26623) as the reference. Then, PVDF membranes were blocked in 3% BSA (prepared in 1× TBST, pH 7.4, same as below) for 1 hour at 20~25°C and were incubated with primary antibodies with desired dilution ratio in 3% BSA/TBST overnight at 4°C. The next day, membranes were rinsed three times for 5 minutes with 1× TBST and were incubated with species-relevant HRP-linked secondary antibodies (1 : 500–1, 000) for one hour at 20~25°C. After being rinsed with TBST, membranes were incubated in an Amersham ECL Prime Western Blotting Detection Reagent (GE Healthcare, RPN2232) or Clarity Max^™^ Western ECL Substrate (Bio-Rad, 1705062). Target bands were clarified using an ImageQuant LAS 400 system (GE Healthcare), and band luminosity was quantified using the inbuilt ImageQuant TL software (Version 7.0 GE Healthcare) and was standardized to GADPH.

### 2.9. Immunofluorescence

As previously described, cells were scoured with PBS three times and were fixed with 4% paraformaldehyde for 1 h at 20~25°C [28]. After overnight incubation with primary antibodies in 5% NDS at 4°C and rinsing of redundant primary antibodies (3 × 10 min with 0.1% Triton X-100 in PBS), cells were incubated for 2 hours at 20~25°C with secondary antibodies diluted in 5% NDS. Coverslips were then flushed with PBS-T (3 × 10 min) and PBS (1 × 10 min) in darkness and then laid down slides using Fluoromount-G (SouthernBiotech, Birmingham, AL). Immunostained preparations were analyzed by confocal microscopy with a Nikon A1R confocal microscope (Nikon Instruments Inc, Melville, NY) to evaluate protein staining.

### 2.10. TUNEL Staining

Cell death was analyzed through terminal deoxynucleotidyl transferase dUTP nick-end labeling (TUNEL) (Roche) as directed by the manufacturer's instructions.

### 2.11. Protein Concentration Analysis and Cell Counting in BALF

After the sacrifice of murine, cooled PBS was injected intratracheally to collect 2 ml of BALF. After centrifugation for 10 minutes at 1500 rpm at 4°C, protein levels in BALF were measured by Bradford Protein Quantification Kit (Beyotime Institute of Biotechnology, China). Wright-Giemsa staining (Beyotime Institute of Biotechnology, China) and a hemocytometer were used to count total cells and different subtypes of hemocytes, including leukocytes, neutrophils, and macrophages [23]. Commercially available ELISA kits (R&D Systems, Minneapolis, MN, USA) were used to estimate the secretion of proinflammatory cytokines (IL-1*β*, TNF-*α*, IL-6, and MCP-1).

### 2.12. mPTP Opening and ATP Production

The opening of mPTP was deemed as a transient attenuation of tetramethylrhodamine ethyl ester fluorescence. Haphazard mPTP opening time was reflected as duration of tetramethylrhodamine ethyl ester (TMRE) fluorescence intensity—its length is half of intensity from initial to residual fluorescence intensity [29]. A firefly luciferase-based ATP assay kit (Beyotime) was used to evaluate cellular ATP content via the tailored protocol.

### 2.13. Bax, Caspase12, and Caspase3 Activity

Bax, Caspase12, and Caspase3 activity (Beyotime, China) was evaluated using commercial kits as previously described [30].

### 2.14. Mitochondrial Membrane Potential Measurement, Mitochondrial Morphology Analysis, and Respiratory Chain Complex Activity Assays

Mitochondrial membrane voltage was evaluated by a JC-1 probe (T131054, Aladdin, Shanghai, China). The mitochondrion selective MitoFluor^™^ stain (Molecular Probes, USA) was applied to locate the mitochondria. The confocal microscope was used to observe the changes of mitochondrial morphology. The activity of complex I, complex II, and complex V was also assessed as previously documented [14].

### 2.15. Malondialdehyde (MDA), Superoxide Dismutase (SOD), and Glutathione (GSH) Assays and Reactive Oxygen Species (ROS)

The MDA content, SOD activity, and GSH concentration were measured following the manufacturer's instructions. ROS was detected with 2′,7′-dichlorofluorescein-diacetate (DCFHDA, Beyotime Institute of Biotechnology, Jiangsu, China) and dihydroethidium (DHE, Invitrogen, San Diego, CA, USA) staining as previously described [31].

### 2.16. Statistics

The Mann–Whitney *U* test was administered to gauge significant discrepancy between means of two independent groups. One-way ANOVA and Student Newman–Keuls test were used to compare discrepancy between three or more groups. The power calculations were utilized to ascertain appropriate sample sizes for all studies in vivo. All data is presented as mean ± SEM. Discrepancy was considered significant when *p* < 0.05.

## 3. Results

### 3.1. NR4A1 Is Increased in LPS-Treated Lung Tissue and Contributes to Acute Lung Injury

**Figure 1:**
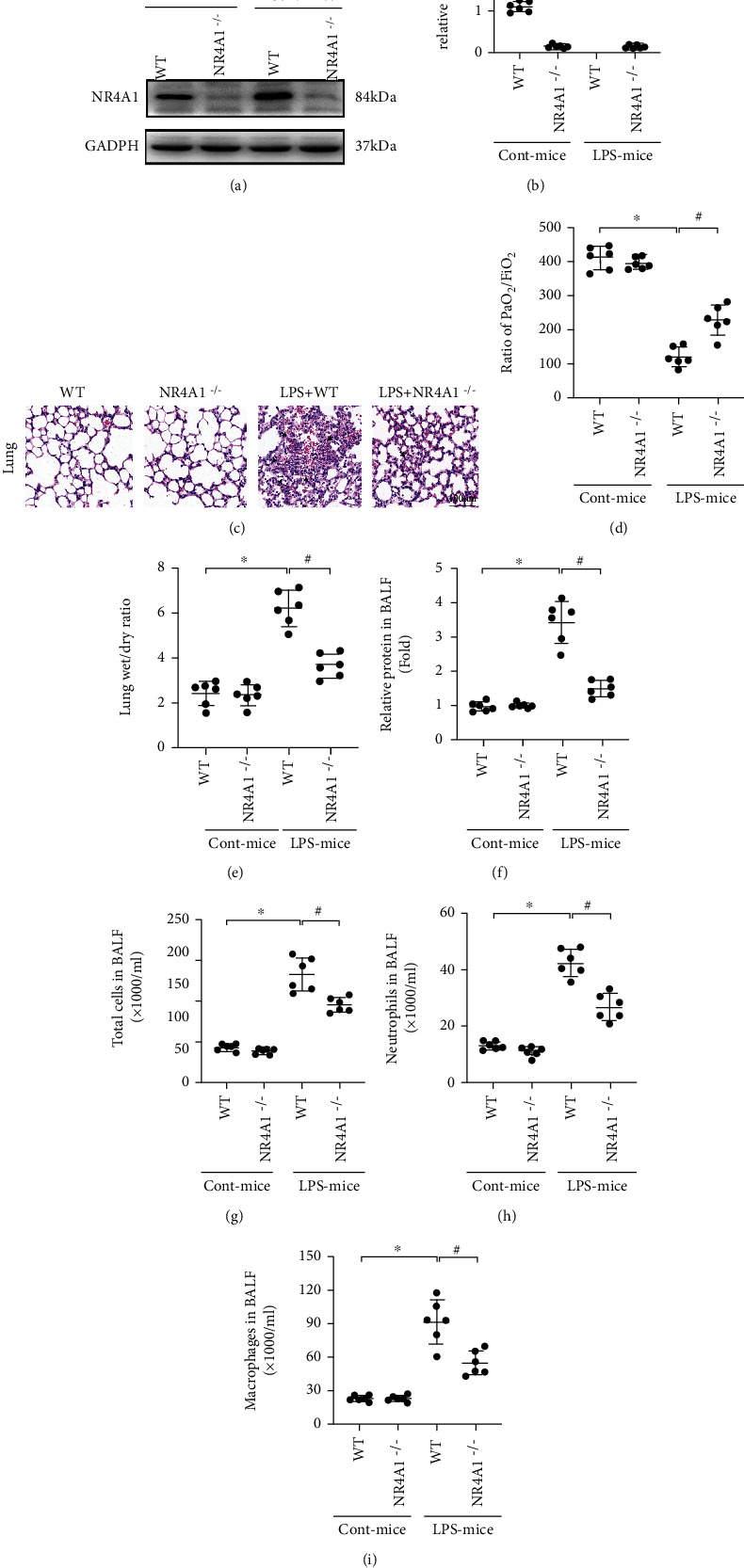
LPS-induced acute lung injury was abolished in response to NR4A1 depletion. (a, b) The change of NR4A1 expression was measured using Western blotting. (c) Pathological changes of lung parenchyma observed by HE staining after induction of acute lung injury. (d) Ratio of PaO_2_/FiO_2_. (e) Lung wet weight to dry weight ratio. (f) Amount of protein in BALF was determined. The number of total cells (g), neutrophils (h), and macrophages (i) in BALF was analyzed to determine lung permeability. Mean ± SEM, ^∗^*p* < 0.05 vs. the WT group; ^#^*p* < 0.05 vs. the LPS group.

Protein analysis was performed to evaluate NR4A1 expression in LPS-induced acute lung injury (Figures 1(a) and 1(b)). Relative to the baseline, NR4A1 expression was upregulated in lung tissues from LPS-treated mice. To explore whether upregulated NR4A1 was associated with LPS-mediated lung damage, NR4A1^−/−^ mice were employed. As shown in Figure 1(c), HE staining revealed inflammatory infiltration, dropsy between alveolus and interstitium, hemorrhagia, and diffused alveolar injury in lung tissues from LPS-treated mice, whereas these alterations were alleviated by NR4A1 ablation. Besides, genetic deletion of NR4A1 improved the index of partial pressure of arterial oxygen (PaO_2_)/percentage of inspired oxygen (FiO_2_) (Figure 1(d)) and normalized the W/D ratio (Figure 1(e)) in the face of LPS insult. Furthermore, LPS overtly evoked accumulation of proteins, total cells, neutrophils, and macrophages in BALF, the effects of which were attenuated by NR4A1 knockout (Figures 1(f) and 1(i)). Taken together, these data indicate that NR4A1 deteriorates lung injury followed LPS treatment.

### 3.2. NR4A1 Deletion Reduces Oxidation Stress and Inflammation Response in LPS-Treated Lung Tissue

**Figure 2:**
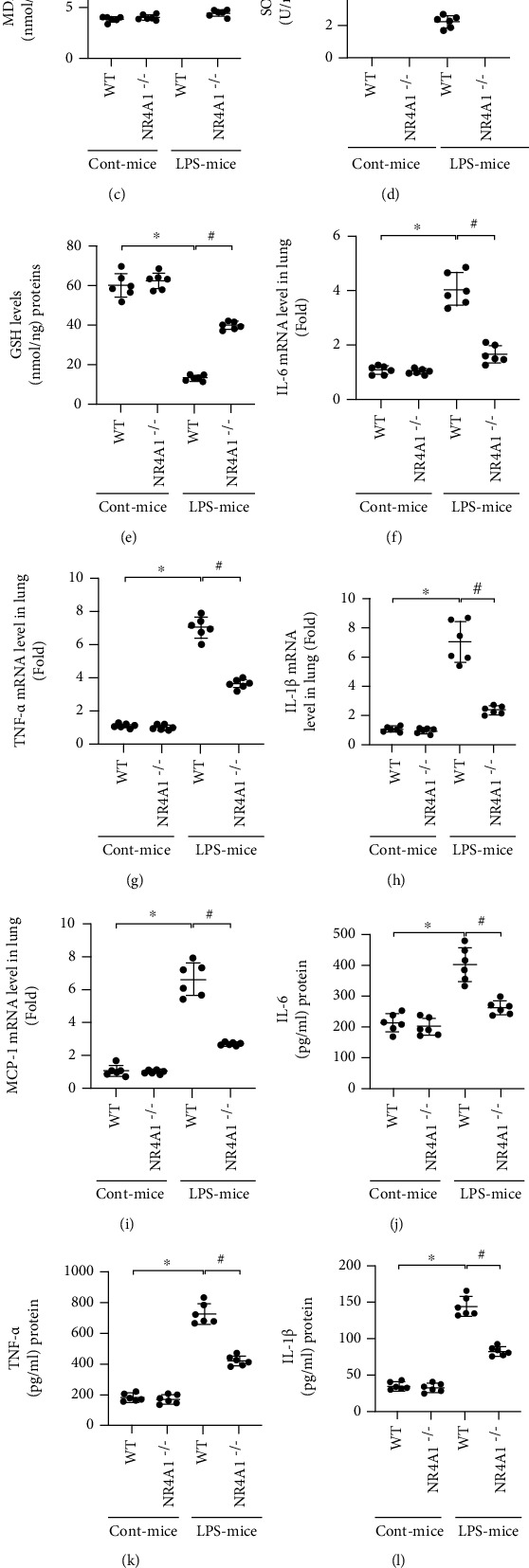
NR4A1 deletion attenuates LPS-mediated oxidative stress and inflammation response in acute lung injury. (a, b) DHE staining was used to detect ROS content. MDA levels (c), SOD activity (d), and GSH levels (e) were also performed in lung tissue. IL-6 (f), TNF-*α* (g), IL-1*β* (h), and MCP1 (i) mRNA levels in murine lung tissues were calculated via RT-qPCR analysis. Protein levels of IL-6 (j), TNF-*α* (k), IL-1*β* (l), and MCP1 (m) were measured by ELISA. Mean ± SEM, ^∗^*p* < 0.05 vs. the WT group; ^#^*p* < 0.05 vs. the LPS+WT group.

Considering the crucial role of oxidation damage in LPS-induced acute lung injury, we further examined whether NR4A1 deletion inhibited oxidative stress in the presence of LPS. DHE staining revealed that ROS was drastically increased in pathological tissue of acute lung injury murine and was decreased to baseline levels in NR4A1-deleted mice (Figures 2(a) and 2(b)). Moreover, LPS evoked MDA generation (Figure 2(c)) and downregulated antioxidant factors, such as GSH and SOD (Figures 2(d) and 2(e)). Nonetheless, these alterations were normalized following NR4A1 deletion.

With respect to inflammation response, LPS significantly upregulated mRNA levels of several cytokines including IL-6, TNF*α*, IL-1*β*, and MCP-l in lung tissues (Figures 2(f) and 2(i)). Interestingly, NR4A1 deletion exerted an anti-inflammatory property, featured by decreased above cytokine levels in the face of LPS challenge. In addition, protein levels of IL-6, TNF*α*, IL-1*β*, and MCP-l in BALF were also increased, as assessed by ELISA (Figures 2(j)–2(m)). As expected, NR4A1 deletion confirmed the LPS-mediated secretion in these proinflammation factors. Overall, these data support a role for antioxidative and anti-inflammatory actions by NR4A1 deletion in LPS-induced acute lung injury.

### 3.3. LPS Promotes Alveolar Epithelium Death through Apoptosis or Necroptosis

**Figure 3:**
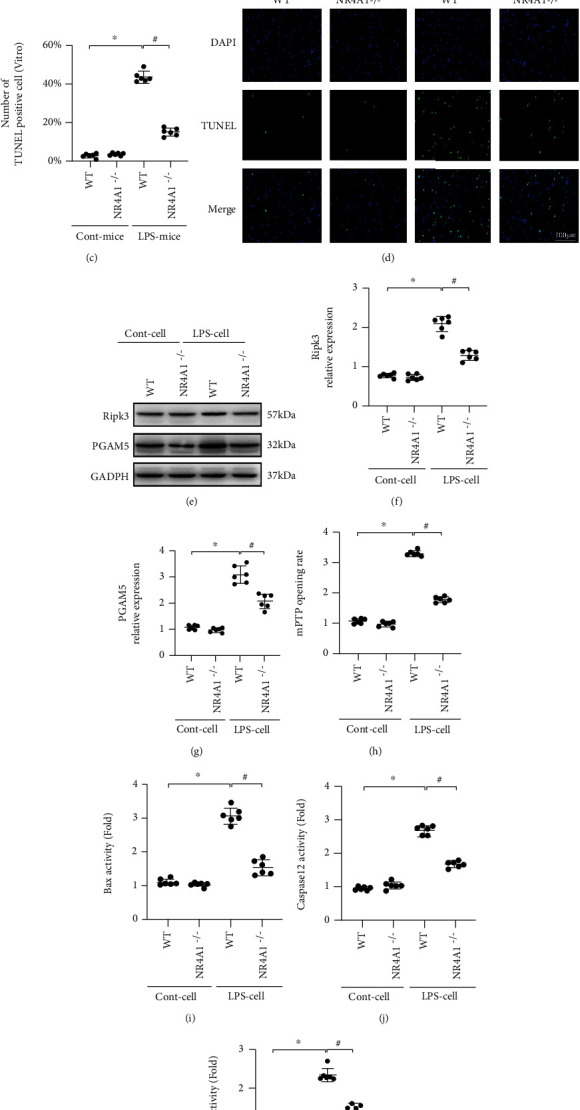
Effect of NR4A1 deletion on LPS-mediated alveolar epithelium death. (a, b) The change of cellular death was detected by TUNEL assay in lung tissue followed by LPS treatment. (c, d) In vitro, NR4A1 deletion significantly reduced TUNEL-positive cells in the face of LPS challenge. (e–g) NR4A1 deletion suppressed accumulation of Ripk3/PGAM5 in alveolar epithelium under LPS challenge. (h) Change in mitochondrial permeability transition pore (mPTP) opening. (i–k) The activities of Bax, caspase-12, and caspase-3 were measured by ELISA. Mean ± SEM, ^∗^*p* < 0.05 vs. the WT group or Ctrl group; ^#^*p* < 0.05 vs. the LPS+WT or LPS group.

ARDS is featured by functional cell loss due to death. To understand whether alveolar epithelium apoptosis or necroptosis is involved in LPS-induced acute lung injury, cell signaling studies were conducted to analyze necroptosis and apoptosis. TUNEL staining depicted that more than 40% of alveolar epithelium was TUNEL-positive upon LPS exposure, and such ratio was dropped to 15% by NR4A1 knockdown (Figures 3(a) and 3(b)). These data suggest that LPS-caused alveolar epithelial death, including apoptosis and necroptosis, was inhibited by NR4A1 deletion, similar with results in vitro studies (Figures 3(c) and 3(d)). Western blot analysis then revealed that proteins related to necroptosis, such as Ripk3 and PGAM5, were upregulated by LPS (Figures 3(e)–3(g)). However, NR4A1 deletion suppressed the accumulation of Ripk3/PGAM5 in alveolar epithelium in the presence of LPS stress. In addition to protein accumulation, mPTP opening served as another marker of necroptosis due to its action in promoting mitochondrial membrane rupture. As shown in Figure 3(h), mPTP opening was increased in alveolar epithelium following LPS stress and such trend was reversed by NR4A1 deletion. Taken together, these data suggest that LPS promoted activation of necroptosis in alveolar epithelium through upregulation of NR4A1.

With regard to apoptosis, proteins were extracted from cells, and ELISA was used to observe alterations of proteins governing cell apoptosis such as Bax and caspase family. Relative to baseline, activities of Bax, caspase-12, and caspase-3 were significantly elevated in LPS-treated cells (Figures 3(i)–3(k)). Interestingly, inhibition of NR4A1 prevented activation of Bax, casapase-12, and caspase-3, denoting a possible role played by NR4A1 in apoptosis in the presence of LPS stress.

### 3.4. Mitochondrial Function Is Disrupted by LPS due to Increased NR4A1

**Figure 4:**
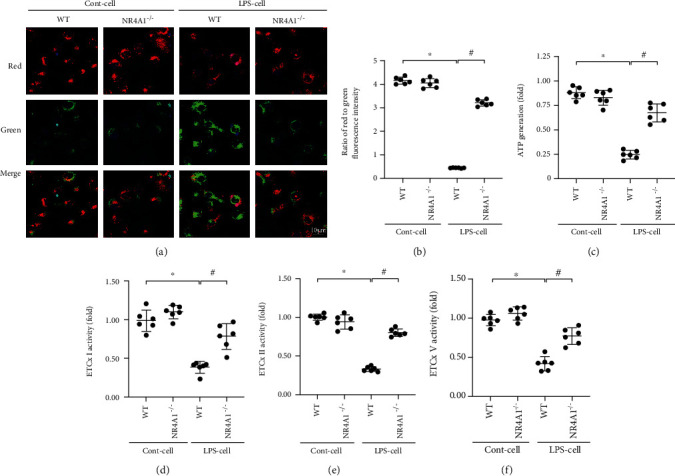
LPS-mediated mitochondrial injury was abolished in response to NR4A1 depletion. (a, b) Changes of mitochondrial membrane potential were detected by JC-1 staining. (c) ATP generation was measured to explore the LPS-induced mitochondrial dysfunction. (d–f) Changes in ETCx I, II, and V activities measured spectrophotometrically. Mean ± SEM, ^∗^*p* < 0.05 vs. the Ctrl group; ^#^*p* < 0.05 vs. the LPS group.

As mentioned above, mitochondrial function as the main energy-producing organelles in mammalian cells and impaired mitochondrial function has been identified as a necessary step to induce acute lung injury. In the present study, at 24 hours following LPS treatment, a markedly lower mitochondrial membrane potential—a marker of mitochondrial damage and bioenergetic impairment—was evident in alveolar epithelium when compared to the control group (Figures 4(a) and 4(b)). However, loss of NR4A1 prevented mitochondrial membrane potential reduction induced by LPS, as witnessed by increased red-to-green fluorescence intensity of JC-1 mitochondrial potential probe. Consistent with these findings, LPS substantially suppressed ATP production, whereas this alteration was reversed by NR4A1 deletion (Figure 4(c)). At the molecular levels, activities of mitochondrial respiratory complex are vital for mitochondrial function and mitochondrial membrane potential maintenance. Interestingly, LPS administration resulted in attenuation of mitochondrial respiratory complex activity whereas this action was detectable in cells by NR4A1 deletion (Figures 4(d)–4(f)). Therefore, these results illustrate that mitochondrial function was sustained by NR4A1 deletion in the face of LPS stress.

### 3.5. NR4A1 Deletion Activates Opa1-Related Mitochondrial Fusion Which Is Required for Mitochondrial Protection

**Figure 5:**
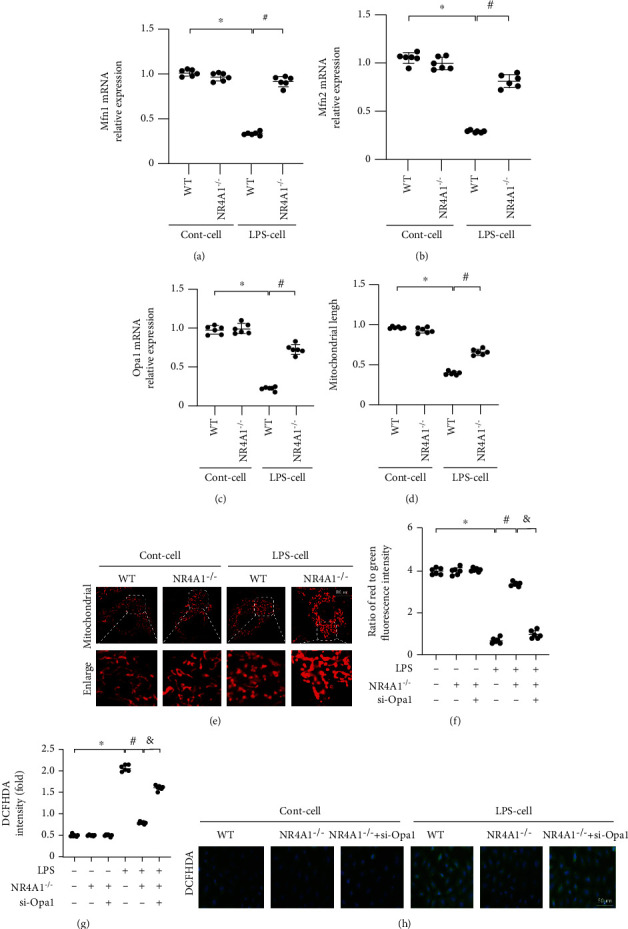
NR4A1 deletion inhibited LPS-induced mitochondrial injury through Opa1-related mitochondrial fission. Mfn1 (a), Mfn2 (b), and Opa1 (c) mRNA levels were evaluated using RT-qPCR. (d, e) Immunofluorescence for mitochondria. Meanwhile, the number of cells with fragmented mitochondria was recorded. (f) JC-1 probe was used to evaluate mitochondrial potential. siRNA against Opa1 was transfected into NR4A1-deleted. (g, h) DCFH-DA staining was used to detect ROS content. ^∗^*p* < 0.05 vs. the Ctrl group. Mean ± SEM, ^#^*p* < 0.05 vs. the LPS group; ^&^*p* < 0.05 vs. the LPS+NR4A1^−/−^ group.

Previous studies have noted that NR4A1 deficiency sustained mitochondrial homeostasis through normalization of mitochondrial dynamics, including mitochondrial fusion and fission. Interestingly, decreased mitochondrial fission has been found to be associated with NR4A1 deletion in ARDS, whereas alteration of mitochondrial fusion in response to NR4A1 deletion remains elusive. In the present study, qPCR analysis demonstrated that transcription of genes associated with mitochondrial fusion including Mfn1, Mfn2, and Opa1 was significantly downregulated under LPS stress (Figures 5(a)–5(c)). Knockdown of NR4A1 promoted the transcription of Mfn1, Mfn2, and Opa1. This finding was also verified through morphological alterations of mitochondria in the face of LPS stress and/or NR4A1 deletion. As shown in Figures 5(d) and 5(e), normal mitochondria under physiological condition exhibit a well-organized network and the average length of mitochondria was around 9.8 *μ*m. Upon LPS stress, mitochondrial network was disrupted, and percentage of fragmented mitochondria was drastically increased. Besides, the average length of mitochondria was reduced to 4.3 *μ*m. Of note, NR4A1 deletion significantly restored mitochondrial length and suppressed ratio of fragmented mitochondria, resulting in improved mitochondrial network structure. In this context, our data indicate that mitochondrial fusion is suppressed by LPS possibly due to downregulated NR4A1.

To understand whether mitochondrial fusion is permissive to NR4A1-offered mitochondrial protection in LPS-treated alveolar epithelium, siRNA against Opa1 was transfected into NR4A1-deleted cells. Then, mitochondrial function was evaluated again. As shown in Figure 5(f), LPS decreased mitochondrial membrane potential and this alteration was attenuated by NR4A1 deletion. However, in NR4A1-deleted cells, Opa1 siRNA failed to sustain mitochondrial potential, suggesting a necessary role for Opa1-mediated mitochondrial fusion in NR4A1-modulated mitochondrial potential stabilization. Besides, cellular oxidative stress, evoked mainly by mitochondrial dysfunction in the presence of LPS stress, was alleviated by NR4A1, whereas this effect was absent in cells transfected with Opa1 siRNA (Figures 5(g) and 5(h)). Therefore, the above data unveil that Opa1-mediated mitochondrial fusion is required for NR4A1 deletion-mediated mitochondrial protection.

### 3.6. Reactivation of PGAM5-Related Necroptosis Attenuates the Prosurvival Effects Afforded by NR4A1 Deletion

**Figure 6:**
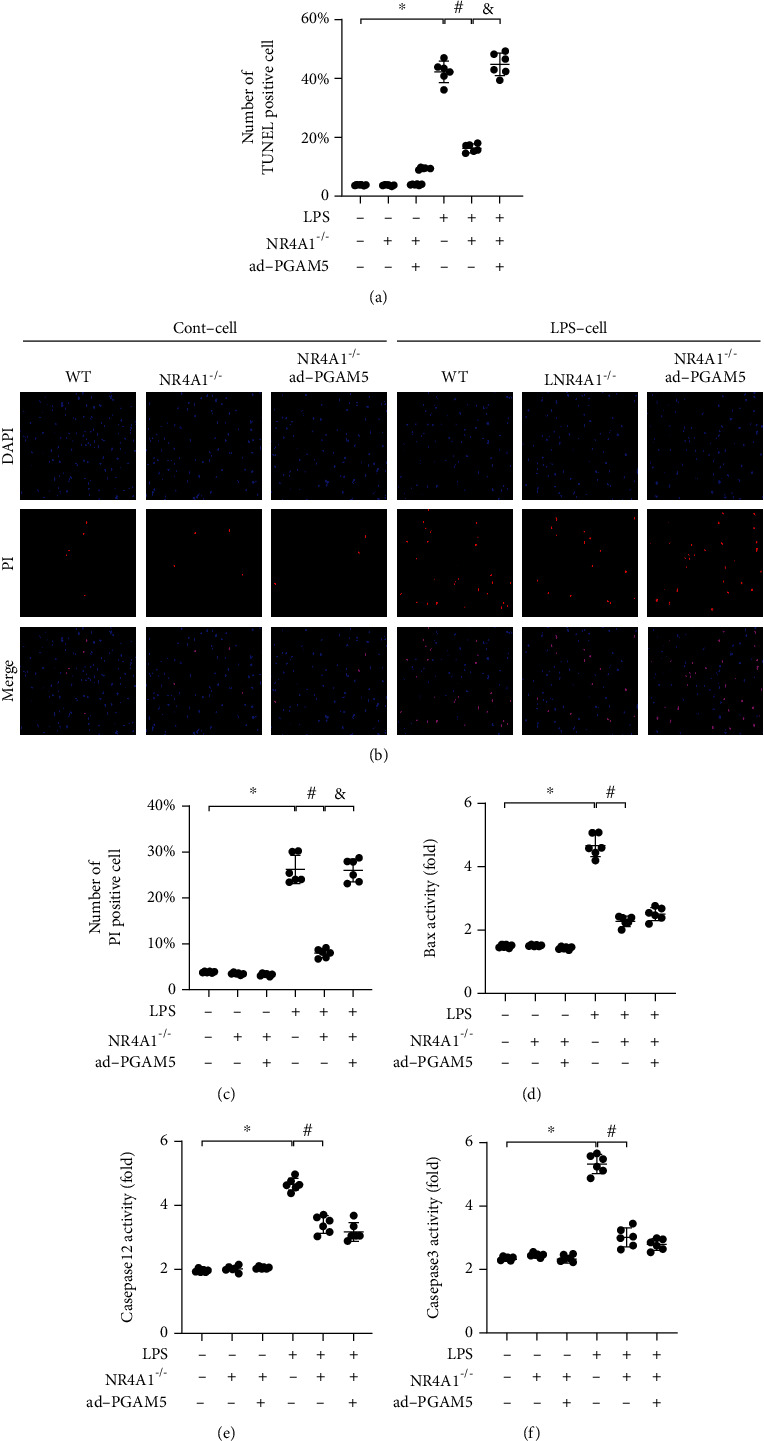
PGAM5 overexpression attenuated the prosurvival effects afforded by NR4A1 deletion. (a) Adenovirus-induced PGAM5 overexpression assay was conducted to reactivate necroptosis in NR4A1-deleted cells. Antiapoptotic effect of NR4A1 deletion was abolished by PGAM5 overexpression in the face if LPS challenge. (b, c) PI staining was applied to further quantify the number of necroptotic cells. Activities of Bax (d), caspase-12 (e), and caspase-3 (f) were measured using ELISA. Mean ± SEM, ^∗^*p* < 0.05 vs. the Ctrl group; ^#^*p* < 0.05 vs. the LPS group, and ^&^*p* < 0.05 vs. the LPS+NR4A1^−/−^ group.

To understand whether NR4A1-mediated necroptosis inhibition contributes to alveolar epithelial survival in the presence of stress, adenovirus-induced PGAM5 overexpression assay was conducted to reactivate necroptosis in NR4A1-deleted cells. Then, alveolar epithelial death was detected. As shown in Figure 6(a), alveolar epithelial death was induced by LPS, manifested by more than 40% TUNEL-positive cells in the LPS-treated group. Although NR4A1 deletion reduced TUNTL-positive cells to ~13%, this action was trivial in cells transfected with PGAM5 adenovirus. Furthermore, PI staining was applied to further quantify the number of necroptotic cells. As shown in Figures 5(b) and 5(c), more than ~28% PI-positive cells were noted in the LPS-treated group, whereas this ratio was dropped to ~8% following NR4A1 deletion. However, in NR4A1-deleted cells transfected with PGAM5 adenovirus, the number of trypan blue-positive cells was increased to ~27%, suggesting that LPS-mediated necroptosis is primarily regulated by NR4A1 in a manner dependent on PGAM5. Bax permeabilizes the outer mitochondrial membrane and induces cell apoptosis via releasing excessive cytochrome c and activating caspase-3. Thus, the effect of NR4A1 on cellular apoptosis was also measured via the Bax, caspase-12, and caspase-3 activity. We noted that PGAM5 overexpression had no effects on Bax, caspase-12, and caspase-3 activity, even though their activities were downregulated by NR4A1 deletion (Figures 6(d) and 6(f)). Therefore, this information suggests that reactivation of PGAM5-mediated necroptosis alleviated the prosurvival effects afforded by NR4A1 deletion.

## 4. Discussion

As an important organ to assure the basic supplementary of oxygen, the lung is also vulnerable to many internal and external factors, leading to the damage of pulmonary stroma and alveoli. Lung diseases are the second most devastating medical issue in the world nowadays, mainly presented as acute lung injury, asthma, COPD, cancer, and ventilator-induced lung injury (VILI) [32]. Among these anomalies, acute lung injury is known as a severe health issue in developing countries accounting for 30% of all-cause mortalities. Mechanistically, the acute lung injury is a clinical respiratory disease, which probably developed into a more complex syndrome, namely, acute respiratory distress syndrome (ARDS). Data from WHO Statistics accounted for ARDS as the leading respiratory death worldwide. Approximately 2 million ICU days and 75 thousand deaths can be credited to ARDS in the US each year. Clinical standards for acute lung injury/ARDS were established in 2012 commonly as known as the “Berlin definition” [33]. Recent studies have noted contribution of oxidative stress in acute lung injury. Due to its particularity, lung tissues localize in a relatively enriched oxygen environment, and it is more likely to produce a large amount of reactive oxygen species in various physiological processes [34]. Oxidative stress is a state in which cells metabolize to produce excess ROS, resulting in damage to DNA, proteins, and lipids [34]. It is related to harmful substances such as superoxide anion free radicals and disproportionation product hydrogen peroxide (H_2_O_2_) generated by mitochondria or enzymatic reaction. The main pathogenic agent of sepsis or acute lung injury is LPS, and LPS first evokes mitochondrial damage, destroys mitochondrial electron transport chain and mitochondria DNA (mtDNA), and promotes excessive ROS production and oxidative phosphorylation inhibition, resulting in increased oxidative stress, cell death, and lung tissue damage [35–37]. In the present study, our data identified the function of NR4A1 as a master of acute lung injury in a mouse model of LPS-induced ARDS. These data convincingly described a role for Opa1-induced mitochondrial fusion and PGAM5-required necroptosis in the development of LPS-mediated acute lung injury.

Mitochondria, the main energy powerhouse of cells, are the potential targets to induce acute lung injury in response to LPS stress. Previous studies found overtly decreased activity of mitochondrial complex after LPS challenge [38]. Other studies have reported that the NF-*κ*B signaling pathway is activated by mitochondrial dysfunction in lung inflammation [39]. In acute liver failure, mitochondrial dynamic impairment and mitochondrial dysfunction have been identified as the critical subcellular pathophysiological processes [40]. In LPS-evoked acute kidney injury, damaged mitochondrial quality control is considered as a main driving force for ROS overproduction and oxidative stress in tubular cells [41]. In septic cardiomyopathy, mitophagy activity is downregulated, and mitochondrial biogenesis is reduced, contributing to cardiomyocyte energy metabolism disorder and myocardial contractile failure [42]. In accordance with these findings, our study also noted damaged mitochondrial, including disrupted mitochondrial structure and decreased mitochondrial function, in LPS-treated lung tissue. In fact, the role of mitochondrial dysfunction in ARDS has been widely examined. For example, acute lung injury is featured by metabolic alterations under mitochondrial regulation [43]. Decreased mitochondrial ATP production and increased mitochondrial ROS generation have been observed LPS-treated lung tissues [13]. Interestingly, one intriguing finding from our present study suggested morphological alterations of mitochondria in LPS-treated lung tissue. Compared to functional disorder, mitochondrial morphological damage seems to be an early sign of mitochondrial damage although this concept has not been confirmed in the acute lung injury. We found that mitochondrial dysfunction is affected by mitochondrial morphological alteration and induction of mitochondrial morphological alteration causes mitochondrial dysfunction in LPS-treated alveolar epithelium. This finding provides a novel insight into the molecular mechanisms behind LPS-induced acute lung injury.

Beside mitochondria fusion, mitochondrial fission, controlled via dynamin-related protein 1 (Drp1) and mitochondrial fission factor (Mff), is responsible for regulating their morphology and number, and the two are generally in a state of dynamic balance. In this study, we confirm that NR4A1 inhibit mitochondrial fusion via reducing transcription of Mfn1, Mfn2, and Opa1. Besides, NR4A1 was confirmed to promote Drp1-related mitochondrial fission in alcohol-related liver disease [44, 45]. Whether NR4A1 affects Drp1 and followed mitochondrial fission in inflammation-related lung injury needs to be performed in subsequent studies.

Unlike apoptosis, necroptosis is a novel type of regulated cell death machinery that can be interrupted through inhibition of Ripk3 or knockdown of PGAM5 [26, 46–48]. In contrast to apoptosis, the role and mechanism of necroptosis in ARDS remain poorly understood. In the present study, both of apoptosis and necroptosis are found to be activated by LPS, indicating that necroptosis may be a neglected issue in the pathogenesis of ARDS. Besides, our results further revealed that LPS-evoked necroptosis is controlled by the NR4A1/PGAM5 signaling pathway. Knockdown of NR4A1 is capable of retarding necroptosis activation whereas this effect was nullified by PGAM5 overexpression. Nonetheless, several issues remain to be addressed. First, it is still unknown if necroptosis and apoptosis interplay with each other during ARDS. Second, whether necroptosis and apoptosis are regulated by a common upstream protein or signaling pathway remains unclear.

**Figure 7:**
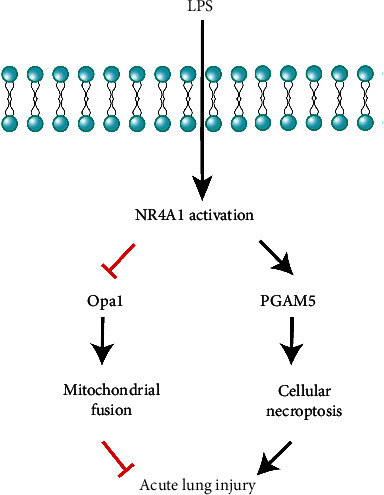
Our results demonstrated a novel role for NR4A1 in LPS-induced acute lung injury through regulating Opa1-mediated mitochondrial fusion and activation of PGAM5-related necroptosis, indicating therapeutic promises of targeting NR4A1 in the treatment of acute lung injury in clinical practice.

Taken together, our results demonstrated a novel role for NR4A1 in LPS-induced acute lung injury through regulating Opa1-mediated mitochondrial fusion and activation of PGAM5-related necroptosis (Figure 7). These findings shed some light towards the therapeutic potential of targeting NR4A1 in the treatment of acute lung injury although more in-depth clinical study is warranted.

## Data Availability

The original data presented in the study are included in the article; further inquiries can be directed to the corresponding authors.
